# FEM-based oxygen consumption and cell viability models for avascular pancreatic islets

**DOI:** 10.1186/1742-4682-6-5

**Published:** 2009-04-16

**Authors:** Peter Buchwald

**Affiliations:** 1 Diabetes Research Institute and the Department of Molecular and Cellular Pharmacology, University of Miami, Miller School of Medicine, Miami, FL, USA

## Abstract

**Background:**

The function and viability of cultured, transplanted, or encapsulated pancreatic islets is often limited by hypoxia because these islets have lost their vasculature during the isolation process and have to rely on gradient-driven passive diffusion, which cannot provide adequate oxygen transport. Pancreatic islets (islets of Langerhans) are particularly susceptible due to their relatively large size, large metabolic demand, and increased sensitivity to hypoxia. Here, finite element method (FEM) based multiphysics models are explored to describe oxygen transport and cell viability in avascular islets both in static and in moving culture media.

**Methods:**

Two- and three-dimensional models were built in COMSOL Multiphysics using the convection and diffusion as well as the incompressible Navier-Stokes fluid dynamics application modes. Oxygen consumption was assumed to follow Michaelis-Menten-type kinetics and to cease when local concentrations fell below a critical threshold; in a dynamic model, it was also allowed to increase with increasing glucose concentration.

**Results:**

Partial differential equation (PDE) based exploratory cellular-level oxygen consumption and cell viability models incorporating physiologically realistic assumptions have been implemented for fully scaled cell culture geometries with 100, 150, and 200 *μ*m diameter islets as representative. Calculated oxygen concentrations and intra-islet regions likely to suffer from hypoxia-related necrosis obtained for traditional flask-type cultures, oxygen-permeable silicone-rubber membrane bottom cultures, and perifusion chambers with flowing media and varying incoming glucose levels are presented in detail illustrated with corresponding colour-coded figures and animations.

**Conclusion:**

Results of the computational models are, as a first estimate, in good quantitative agreement with existing experimental evidence, and they confirm that during culture, hypoxia is often a problem for non-vascularised islet and can lead to considerable cell death (necrosis), especially in the core region of larger islets. Such models are of considerable interest to improve the function and viability of cultured, transplanted, or encapsulated islets. The present implementation allows convenient extension to true multiphysics applications that solve coupled physics phenomena such as diffusion and consumption with convection due to flowing or moving media.

## Background

Type 1 (insulin-dependent or juvenile-onset) diabetes mellitus (T1D) is an autoimmune disease resulting in the destruction of the insulin-producing pancreatic *β*-cells and requiring continuous glucose monitoring and insulin treatment. Chronic and degenerative complications still occur in a considerable fraction of patients. Since transplantation of pancreatic islet cells can normalize metabolic control in a way that has been virtually impossible to achieve with exogenous insulin, it is being explored, in a selected cohort of patients, as an experimental T1D therapy [1,2]. Because of the life-long immunosuppression required, it is currently limited to the most severe forms of diabetes, and, in the US, is currently conducted at several centres under an IND (Investigational New Drug) application. Due to improved islet preparation techniques and the availability of more effective immunosuppressive regimens [3] such as those of the so-called Edmonton protocol [4], results are improving continuously [1,2]. Nevertheless, despite all the progress in islet transplantation and in the development of bioartificial pancreas-type devices [5], the three main critical issues that need to be solved still remain those related to biocompatibility, oxygen supply limitations, and prevention of long-term immune rejection [6].

As a standard practice, islets are usually cultured for up to two days before being transplanted [7,8] because this allows the islets to recover from the isolation-induced damage and also makes possible the recipient's travel to the transplantation site, the start of the immunosuppression before transplantation, and the assessment of the quality and safety of the islets. Short-term culture may also reduce the immunogenicity of islets [7]. However, the survival and functionality of these islets that lost their vasculature during the isolation process and have to rely on gradient-driven passive diffusion is often seriously affected by hypoxia during culture or immediately following transplantation. Hence, the spatio-temporal modelling of oxygen consumption of pancreatic islets (and of other tissues) is an important general goal in itself, but it is of particular interest for the development of improved islet culture and bioartificial pancreas-type devices (with encapsulated or non-encapsulated islets).

Pancreatic islets are structurally well-defined spheroid-like cell aggregates of about 1500–2000 cells and diameters of about 150 *μ*m (range: 50–500 *μ*m) [9,10] that contain the endocrine cells of the pancreas (*α*, *β*, *γ*, and PP-cells) whose main role is to secrete hormones that regulate blood glucose levels. An islet with a diameter of 150 *μ*m is considered as standard to convert islet mass into islet equivalents (IEQ) [9]. A healthy human pancreas contains, on average, around one million islets. Islets possess an extensive intra-islet vasculature, which is needed to supply oxygen and nutrients and to remove metabolic waste products – especially in their inner core [11-13]. Islets have a high blood perfusion: they receive around 10–20% of the total blood flow of the pancreas despite representing only about 1–2% of its weight [14-16]. During islet isolation and culture, this vasculature gets disrupted so that islets are avascular and perfusion of the core is compromised. Hence, cultured or encapsulated (immune-isolated) islets, as well as transplanted islets during the initial few days of transplantation have to depend on the passive diffusion of oxygen and nutrients from the periphery, which limits the oxygen and nutrient supply in the inner core of islets, especially larger islets, and can ultimately lead to hypoxia and cell death [15,17]. Because of these hypoxia-related problems, current islet culture techniques require low surface coverage, and, hence, the use of up to thirty or more flasks per human pancreas (i.e., ~20,000–30,000 IEQ in 30 mL of medium per flask corresponding to 100–200 IEQ/cm^2 ^and a flask surface utilization of only 2–3%) [7,18-20]. This is a considerable hindrance both for research settings and for clinical applications. Consequently, various attempts are being made to enhance oxygenation, for example, by use of silicone rubber membranes [20-23] due to their high oxygen-permeability [24] or by use of bioreactors with rocking plates and wave-induced agitation [25,26]. Exploratory computational models for some of these will be presented here.

Oxygen diffusion limitations in tissue or in culture media are usually far more severe than for glucose [27,28] because even if oxygen is typically consumed at approximately the same rate as glucose (on molar basis) and has a three-four-fold higher diffusion coefficient, this is more than offset by the differences in solubility since oxygen solubility in aqueous media is much lower than that of glucose: around 0.2 mM vs. 5–10 mM (assuming physiologically relevant conditions) [28]. Compared to many other cell cultures or cell transplants, pancreatic islets are particularly susceptible due to their relatively large size, large metabolic demand, and increased sensitivity to hypoxia. Hence, there is a keen interest to model oxygen consumption in non-vascularised islets and to use the acquired information to improve viability *(i) *in culture, *(ii) *immediately following transplantation, or *(iii) *under immune-insulating encapsulation. In the islet field, various models have already been explored, mainly for immunoisolated (encapsulated) islets [27,29-31], and they can also be extended to model tissue oxygenation in other cases of interest such as, for example, during pancreas preservation [32] or in cell devices with oxygen-permeable silicone membranes [20-23]. Similar models for other, e.g., cardiac tissue have also been explored [33], and oxygenation models based on various approaches for certain micro-vascularised tissues have also been published [34,35]. However, essentially all of them incorporated only models of diffusive transport. The approach described here has the advantage that it allows the relatively easy coupling of diffusion and convection models to computational fluid dynamics and other application modes making possible true multiphysics models for more complex cases such as, for example, those with moving media of varying glucose concentrations; perifusion devices with pump-driven flow will be discussed here as one possible application. To the author's knowledge, this is the first time that cellular-level calculations are done for both 2D and 3D geometries in a true multiphysics FEM-based implementation, that the corresponding animations of hypoxia-related cell death are generated and submitted for Web-based publication, and that the glucose-dependence of the oxygen consumption of pancreatic islets is incorporated in a model.

## Methods

### Computational model

A finite element method (FEM) based approach was used as implemented in COMSOL Multiphysics 3.4 (formerly FEMLAB) (COMSOL Inc., Burlington, MA). FEMs represent a numerical technique designed to find approximate solutions of general partial differential equations (PDE) based problems and are well-suited for complex geometries or varying domains since they rely on 'discretization' of the problem, i.e., the geometry is partitioned into small units of a simple shape (e.g., triangles for 2D and tetrahedrons for 3D subdomains) [36].

### Oxygen diffusion and consumption

Diffusion was assumed to be governed by the generic diffusion equation in its nonconservative formulation (incompressible fluid) [37]:

(1)

where, *c *denotes the concentration [mol·m^-3^] and *D *the diffusion coefficient [m^2^·s^-1^] of the species of interest (here, oxygen), *R *the reaction rate [mol·m^-3^·s^-1^], **u **the velocity field [m·s^-1^], and ▽ the standard *del *(*nabla*) operator, [38]. For oxygen consumption, a Michaelis-Menten-type consumption rate (*R *< 0) was assumed as customary in current literature [27,39]:

(2)

Here, *R*_max _is the maximum oxygen consumption rate,  the Michaelis-Menten constant corresponding to the oxygen concentration where consumption drops to 50% of its maximum, *C*_cr _is the critical oxygen concentration below which necrosis is assumed to occur after a sufficiently long exposure, and *δ *a step-down function to account for the ceasing of consumption in those parts of the tissue where the oxygen concentration fell below a *C*_cr _critical concentration. Consensus estimates of various parameters available from the literature were used. Oxygen in aqueous solutions obeys Henry's law rather well; i.e., its (mole fraction) solubility () is essentially proportional to the partial pressure of oxygen () in the surrounding media, [40]. For the present exploratory calculations, *c*_amb _= 0.200 mol·m^-3 ^(mM) was assumed for surfaces in contact with atmospheric oxygen. With an oxygen solubility coefficient of *α *= 1.45 × 10^-3 ^mol·m^-3^mmHg^-1 ^(35°C) [41], this roughly corresponds to a partial pressure  of 140 mmHg. A maximum oxygen consumption rate *R*_max _(per unit islet volume) of 0.034 mol·s^-1^·m^-3 ^was used in all calculations. With a standard islet of 150 *μ*m diameter (and islet equivalent IEQ volume *V*_IEQ _of 1.77 × 10^-12 ^m^3^), this corresponds to a consumption rate (per islet) of *R*_max _= 0.06 × 10^-12 ^mol·s^-1^/islet; both values being in the range of those measured and used in various works [20,23,29,30,32,42-46]. As Michaelis-Menten constant,  = 1.0 × 10^-3 ^mol·m^-3 ^(1 *μ*M) was assumed, corresponding to  = 0.7 mmHg – similar to the frequently used 0.44 mmHg value [23,27,29,32] or even to that determined originally for mitochondria [39]. A step-down function, *δ*, was also added to account for necrosis and cut the oxygen consumption when the concentration fells below a critical value, *C*_cr _= 1.0 × 10^-4 ^mol·m^-3 ^(corresponding to  = 0.07 mmHg; comparable with the commonly used 0.10 mmHg [23,27,32]). COMSOL's smoothed Heaviside function with a continuous first derivative and without overshoot flc1hs [47] was used as step-down function, *δ *(*c*) = flc1hs(*c*-1.0 × 10^-4^, 0.5 × 10^-4^). In the dynamic model (perifusion chamber), oxygen consumption was allowed to also vary as a function of the local glucose concentration, *c*_gluc_, to account for the increased metabolic demand of insulin production at higher glucose concentrations. As a first modelling attempt, this was done by introduction of an additional Michaelis-Menten-type dependency on *c*_gluc_:

(3)

The corresponding constants were selected so as to allow an approximate doubling when going from low (3 mM) to high (11 mM) glucose concentration (*C*_MM, luc _= 8 mol·m^-3^, *φ *= 3.67). For the diffusion coefficient of oxygen in aqueous media,  = 3.0 × 10^-9 ^m^2^·s^-1 ^was assumed; a reasonable approximation for O_2 _diffusion in water at 37°C considering the commonly accepted value of 2.4 × 10^-9 ^m^2^·s^-1 ^at 25°C [41] and a measured value of 3.1 × 10^-9 ^m^2^·s^-1 ^at 45°C or fitted diffusivity equations such as the Wilke-Chang or Othmer-Thakar estimates for diffusion coefficient in aqueous solutions [48]. For the diffusion coefficient of oxygen in tissue,  = 2.0 × 10^-9 ^m^2^·s^-1^, was assumed; slightly less than in water and the same value that was used by Radisic, Vunjak-Novakovic and co-workers [33]. Avgoustiniatos and co-workers have recently determined a somewhat lower value for the effective diffusion coefficient of oxygen in rat pancreatic islets (1.3 × 10^-9 ^m^2^·s^-1^) [44]. The same value was used for the diffusion coefficient in silicone since it was within the range of measured values [24,49].

### Fluid dynamics

In the more complex cases where true multiphysics models were needed, the convection and diffusion model of eq. 1 was coupled to a fluid dynamics model. For fluid dynamics, the incompressible Navier-Stokes model for Newtonian flow (constant viscosity) was used to calculate the velocity field **u **that results from convection [37,50]:

(4)

Here, *ρ *denotes density [kg·m^-3^], *η *viscosity [kg·m^-1^·s^-1 ^= Pa·s], *p *pressure [Pa, N·m^-2^, kg·m^-1^·s^-2^], and **F **volume force [N·m^-3^, kg·m^-2^·s^-2^]. The first equation is the momentum balance; the second one is simply the equation of continuity for incompressible fluids. For cases where convective flow was also allowed in the model, an essentially aqueous media at body temperature was assumed as a first estimate: *T*_0 _= 310.15 K, *ρ *= 993 kg·m^-3^, *η *= 0.7 × 10^-3 ^Pa·s, *c*_p _= 4200 J·kg^-1^K^-1^, *k*_c _= 0.634 J·s^-1^m^-1^K^-1^, *α *= 2.1 × 10^-4 ^K^-1^.

### Geometry and boundary conditions

For the present exploratory models, fully scaled realistic 2D and 3D geometries have been used with spherical islets of 100, 150, and 200 *μ*m diameters placed in millimeter-sized device models. COMSOL's predefined 'Extra fine' and 'Fine' mesh size was used for meshing of 2D and 3D geometries, respectively resulting in meshes with 5–10,000 elements in 2D and 150,000 elements in 3D. In the convection and diffusion models, the following conditions were assumed: insulation/symmetry, **n**·(-*D*▽*c*+*c***u**) = 0, for side walls, continuity for islets, and fixed concentration (*c *= *c*_amb_) for liquid surfaces in contact with exterior media (top). For the case of diffusion through a membrane, a membrane/media partition coefficient *K*_p _= *c*_membr_/*c *was built into the model for oxygen through a special boundary condition using the stiff-spring method [51]. An additional, separate concentration variable *c*_2 _was added for the membrane (with a corresponding application mode), and to maintain continuous flux at the interface, an inward flux boundary condition was imposed along the membrane-fluid boundary with *υ *(*c*_2 _- *K*_p_*c*) and *υ *= 10,000 m·s^-1^. In the incompressible Navier-Stokes models, no slip (**u **= 0) was assumed along all surfaces corresponding to liquid-solid interfaces. For the perifusion chamber, a parabolic inflow velocity profile, 4*v*_in_*s*(1-*s*), was used on the inlet (*s *being the boundary segment length) and pressure, no viscous stress with *p*_0 _= 0 on the outlet.

### Implementation

All models were implemented in COMSOL Multiphysics 3.4 and solved as time-dependent problems up to sufficiently long final times to reach steady state allowing free or intermediate time-steps for the solver. Computations were done with the UMFPACK direct solver as linear system solver on a Dell Precision 690 PC with a 3.2 GHz CPU running Linux.

## Results

### Standard culture model

**Figure 1:**
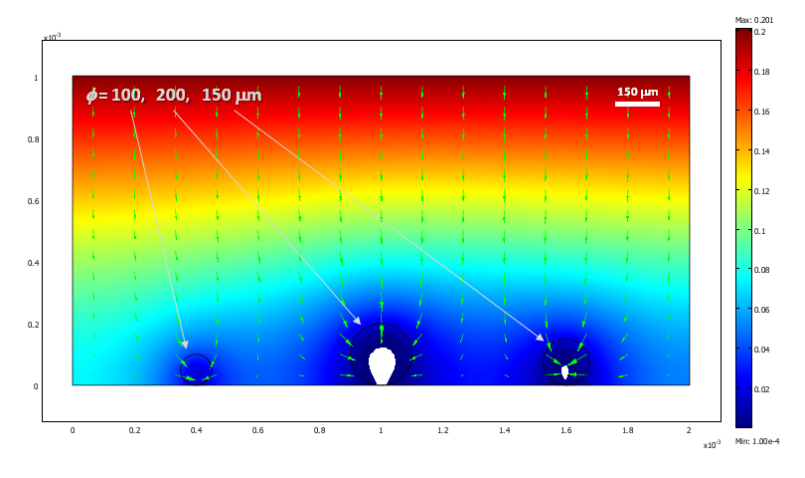
**Calculated oxygen concentration for three islets (with diameters of *ɸ *= 100, 150, and 200 *μ*m) in standard culture conditions after steady state conditions have been reached (*h *= 1 mm assumed) presented as a colour-coded surface with red corresponding to higher and blue to lower concentrations**. Green arrows represent oxygen flux. Areas with oxygen concentrations below a critical value ( < 10^-4 ^mol·m^-3^), where hypoxia is predicted to result in necrosis after a sufficiently long exposure, are coloured in white.

**Figure 2:**
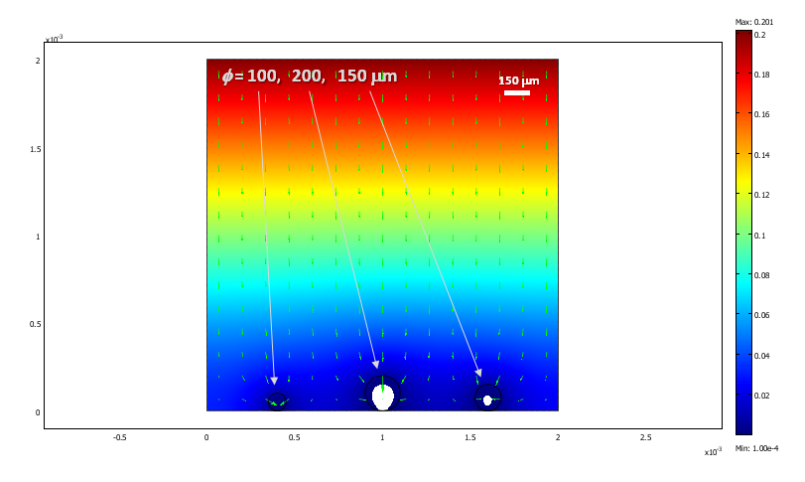
**Calculated oxygen concentration for three islets in conditions similar to Figure 1, but covered with a deeper media (*h *= 2 mm assumed) resulting in decreased oxygenation**.

**Figure 3:**
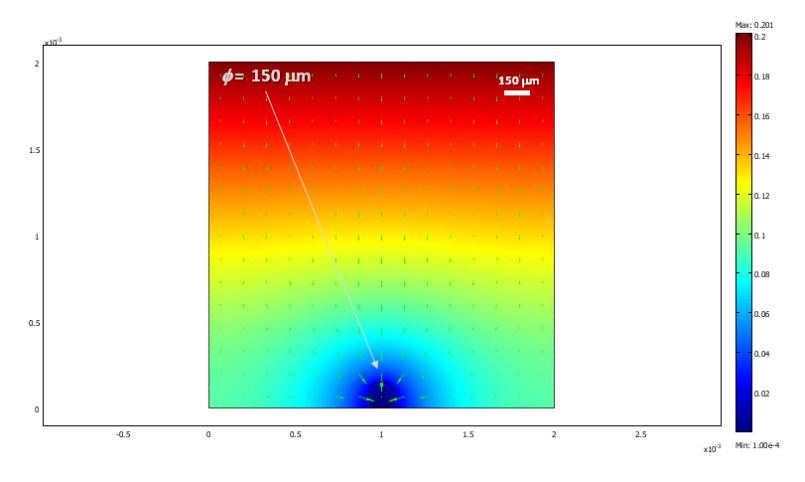
**Calculated oxygen concentration for a single (*ɸ *= 150 *μ*m) islet for the same conditions of Figure 2**.

The oxygen distribution obtained for a two-dimensional cross section of three differently sized islets in a traditional culture model, after steady state conditions are reached, is shown in Figure 1 with a corresponding animation (time-scale in seconds) shown in additional file 1. Since these are 2D cross-sections, the 'islets' here in fact correspond to strings and not spheres; hence, Figure 1 roughly corresponds to a 3D culture density of about 1,600 IEQ/cm^2 ^(i.e., a surface utilization of ~20%). Under these conditions, larger islets are predicted to have necrotic cores, a problem that is not present in smaller islets. The percent of cross-sectional areas predicted in this example to be below the critical oxygen threshold were around 25%, 5%, and 0% for the islets with diameters of 200, 150, and 100 *μ*m, respectively. Overall, calculations are in good agreement with various experimental observations of cultured islets (see Discussion). Obviously, oxygenation can be improved by lowering the density of the consuming tissue, by reducing the diffusion path in the media, or by increasing the outside oxygen concentration. For example, the same three islets are predicted to have larger necrotic portions if the media height is larger (2 mm), and, hence, the diffusion path of the oxygen from the top is also larger (percent areas predicted to be below the critical oxygen threshold were around 50%, 30%, and 0% for the islets with diameters of 200, 150, and 100 *μ*m, respectively) (Figure 2, additional file 2). Actual standard cultures use lower densities (100–200 IEQ/cm^2^) [18-20], and indeed a single standard islet (*ɸ *= 150 *μ*m) in the same 2D culture model, which would correspond to a lower cell density of ~500 IEQ/cm^2^, can survive without any necrosis of its core even in this deeper media (Figure 3, additional file 3).

**Figure 4:**
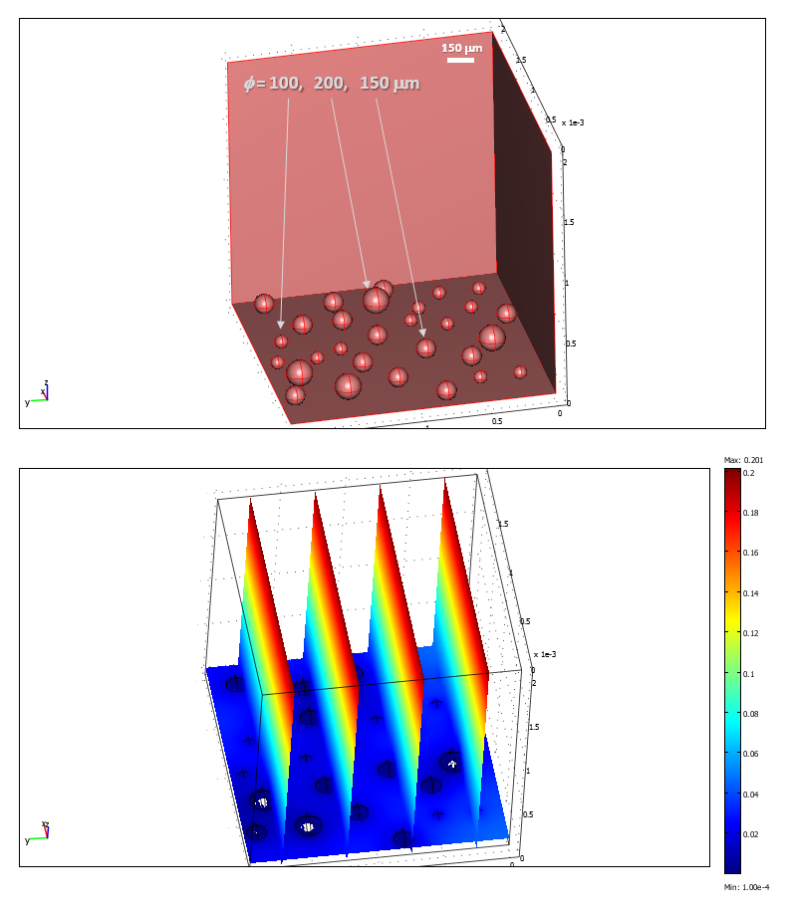
**Calculated oxygen concentrations (bottom) in a three-dimensional islet culture model (top) with differently sized islets (*ɸ *= 100, 150, and 200 *μ*m) randomly distributed at a density of approximately 600 IEQ/cm^2^**. A corresponding time-dependent animation is shown in additional file 4.

A true three-dimensional simulation of such a culture was also run; however, such calculations are more difficult to implement and are considerably more time consuming to run as they contain many more mesh elements. Results obtained for a representative case with randomly distributed islets at a density of approximately 600 IEQ/cm^2 ^and covered by 2 mm of media are shown in Figure 4 with a corresponding animation shown in additional file 4. In these conditions, islets up to about standard islet size (*ɸ *= 150 *μ*m) show essentially no necrosis, but the larger ones show some central necrosis. For example, the larger islets here (*ɸ *= 200 *μ*m) were predicted to have ≈ 10% of their volume as necrotic.

### Oxygen-permeable membrane bottom culture

**Figure 5:**
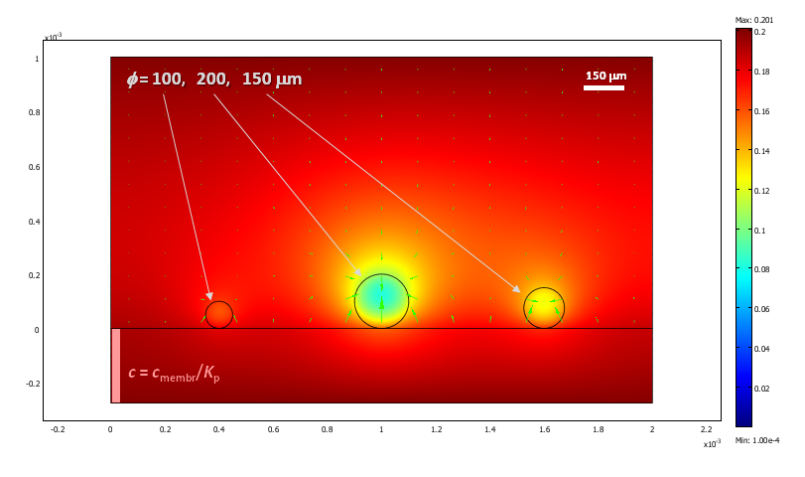
**Calculated oxygen concentration for the three islets of Figure 1 for the same conditions, but with a device with an oxygen-permeable bottom membrane**. Actual oxygen concentrations in the membrane are higher than in the media but are shown here after rescaling with the corresponding the partition coefficient (*K*_p _= *c*_membr_/*c*).

A model of a similar islet culture, but having oxygen-permeable bottom membranes was also explored as such devices are one of the possibilities being investigated to increase the oxygenation of cell cultures in general and islet cultures in particular. Calculations were performed assuming a 0.275 mm thick membrane with ten-fold higher oxygen solubility than water. As Figure 5 and additional file 5 show, much better oxygenations can indeed be achieved with such membranes even at high islet densities in agreement with experimental observations [20,22,23]. All regions of the islets considered were predicted to have oxygen concentrations well above critical levels.

### Perifusion chamber with flowing media

**Figure 6:**
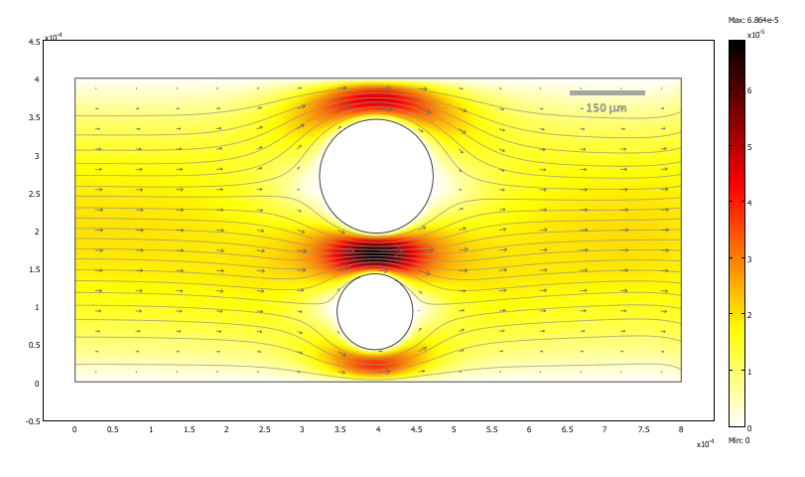
**Velocity field of the fluid flow in a perifusion chamber model with two islets at steady state conditions shown as a colour-coded surface (darker colors corresponding to higher velocities)**. The direction of the velocity field for the flow of the perifusion media is also shown by gray arrows and streamlines.

**Figure 7:**
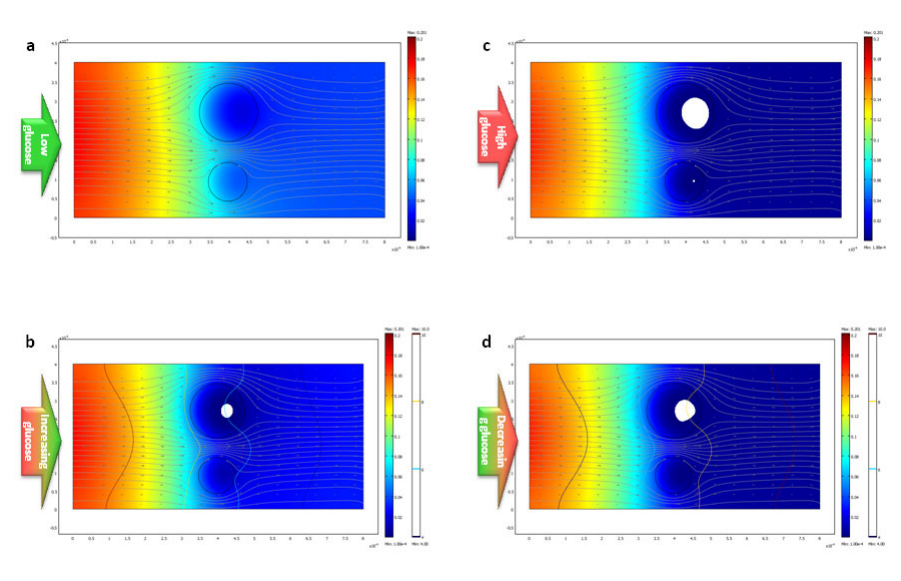
**Calculated oxygen concentrations in a perifusion chamber model with two islets with media flowing from left to right**. Graphics shown correspond to cases of low (3 mM) incoming glucose (**a**), increasing glucose concentration (**b**; note contour lines of the glucose gradient), high (11 mM) incoming glucose (**c**), and decreasing glucose concentration (**d**). See additional file 6 for a corresponding animation.

Finally, a true multiphysics model incorporating both diffusion and convection due to flow was implemented to simulate oxygen consumption in a perifusion chamber model with two islets and moving media; such devices are now frequently used for the dynamic assessment of islet quality and function. As a more realistic model of the dynamics of oxygen consumption, the oxygen consumption of islets was assumed to increase with increasing glucose concentration due to the increased metabolic demand [46,52-55]. As a first, exploratory model, an approximate doubling of the consumption rate was assumed when going from low (3 mM) to high (11 mM) glucose concentration (eq. 3, which at high  would correspond to *R*_max _increasing from 0.034 to 0.074 mol·s^-1^·m^-3^). Figure 6 shows the velocity field of the flowing (incompressible) media; obviously, flow velocity has to increase were the cross section is constrained by the presence of islets to maintain a constant flux. Calculated oxygen concentrations are shown in Figure 7 at various incoming glucose concentrations (with a corresponding animation in additional file 6). At low glucose concentration (3 mM), the islets considered show no necrosis despite the relatively large seeding density because the flowing media can provide better oxygenation (Figure 7a). After the glucose concentration is increased (Figure 7b), the higher metabolic demand is predicted to result in falling of the oxygen concentrations below the critical threshold in certain regions, especially in larger islets (Figure 7c), which might result in necrosis if sufficiently prolonged. If the increased demand lasts for only a relatively limited time, part of the damage might be reversible as the glucose concentration is decreased (Figure 7d).

## Discussion

### Oxygen consumption model

All models implemented here assumed that oxygen consumption takes place only within islet tissues and follows a Michaelis-Menten-type kinetics (eq. 2) that, at non-elevated glucose concentrations, plateaus at a maximum consumption rate *R*_max _(per unit islet volume) of 0.034 mol·s^-1^·m^-3^. This per volume value is similar to that used by Avgoustiniatos and co-workers (0.034 mol/s/m^3 ^[44]; 0.050 mol/s/m^3 ^[23]) and Tilakaratne and co-workers (0.046 mol/s/m^3^) [29]. As a per islet value (0.06 × 10^-12 ^mol·s^-1^/islet), it is similar to that assumed by Dulong and Legallais (0.063 × 10^-12 ^mol·s^-1^/islet) [30,42]; somewhat less than that assumed by Papas, Avgoustiniatos, and co-workers (0.127 × 10^-12 ^mol·s^-1^/islet [20,32]; 0.074 × 10^-12 ^mol·s^-1^/islet [43]); and slightly larger than those measured recently in various settings by Sweet and co-workers (e.g., 0.025–0.048 × 10^-12 ^mol·s^-1^/islet at 3 mM basal- or 20 mM high glucose [45,46]). Converted to a per cell value (3.0 × 10^-17 ^mol·s^-1^/cell), it is also in general agreement with values observed with other high-demand cells [21]. This consumption rate (0.034 mol·s^-1^·m^-3^) means that each volume unit of islet needs about 70 times its volume daily in oxygen as gas (0.034 mol/s/m^3 ^× 24·3600 s/day × 0.02478 m^3^/mol). For comparison, the average respiration rate of a human (16 respiration/min each of ~0.5 L, 4% of which is oxygen consumed) gives an approximate oxygen consumption of 0.3 L/min [56], which means about 500 L/day, i.e., about 6–7 times its volume as living organism. Hence, considering that islets are metabolically high-demand cells receiving about 10 times higher blood flow than their surrounding tissue in the pancreas, this oxygen consumption rate is a realistic first estimate.

The Michaelis-Menten-type consumption rate assures that at very low O_2 _concentrations, where cells only try to survive, oxygen consumption decreases with the available concentration, . Furthermore, a step-down function *δ *was also incorporated into the model to account for necrosis (cell death) and eliminate the oxygen consumption of those tissues where  fell below a critical value, *C*_cr_, and could cause cell death due to hypoxia after a sufficiently prolonged exposure. In general, islets seem to show a size-distribution well described by a Weibull distribution (often used as Rosin-Rammler distribution for particle size), , with most islets having smaller diameters (~50 *μ*m), but the bulk of the volume being contributed by larger (*ɸ *= 2*r *= 100–200 *μ*m) islets [10,57-59]; hence, islets with diameters of 100, 150, and 200 *μ*m were selected as representative here (especially since larger islets are of more interest as hypoxia is more likely to be a problem for them).

### Standard culture model

Results obtained here (Figure 1, 2, 3, 4) are in good overall agreement with various experimental observations indicating that when isolated islets are cultured for 24–48 h in normoxic culture conditions, large islets show central necrosis, which becomes much more severe after exposure to hypoxic culture conditions [15]. As a first estimate, even the size of the necrotic core as measured for rat islets by Vasir and co-workers [15] or by MacGregor and co-workers [60] is well predicted suggesting that these exploratory models provide reasonable quantitative estimates and not just qualitative fit. Results also confirm that in traditional cultures, very low culture densities are needed to ensure viability of the core of larger islets justifying the current standard practice (100–200 IEQ/cm^2 ^corresponding to a surface utilization of only 2–3% [7,18-20]). 3D models are considerably more difficult to implement and time-consuming to run than 2D models; nevertheless, one 3D simulation was performed (Figure 4, additional file 4) to validate the 2D simulations. Similar results were obtained – compare, for example, Figure 3 and Figure 4; in both cases, standard islets (*ɸ *= 150 *μ*m) showed only very minimal central necrosis (<1%) at densities of 5–600 IEQ/cm^2 ^and a media height of 2 mm. Figure 4 also confirms that agglomeration resulting from non-uniform distribution can be a problem as necrotic regions are larger in islets that have close neighbours.

It should be noted that in all these models, instantaneous death for tissues was assumed as soon as the local  values fell below the critical threshold. Hence, while these models are ultimately realistic at steady state, the time-scales, which are shown in seconds in all animations, are probably not, since, under critical conditions, real islets and cells can probably shut down their metabolism more effectively and can survive for some time before irreversible death occurs; more realistic models that can also account for the hypoxia exposure time will be developed in the future. Mammalian cells have developed various mechanisms to survive acute and even prolonged hypoxia [61]. For example, a brief (10 min) ischemic preconditioning might even improve islet cell recovery after cold preservation [62]. On the other hand, there are certainly additional inter-cellular danger- or death-related signals that are not taken into account by the present simplified, oxygen diffusion only models.

### Oxygen-permeable membrane bottom culture

As illustrated by Figure 1, 2, 3, 4, devices with enhanced oxygenations are needed for more efficient islet culture. Use of cell culture devices with oxygen-permeable membrane bottoms is one of the most promising alternatives that are being explored toward this goal [20,22]. Silicone rubber-based membranes are a preferred choice due to their high oxygen-permeability [24]. The solubility of oxygen in such silicone-based materials is also much higher than in water being, for example, around 0.3 cm^3^(STP)·cm^-3^·atm^-1 ^in silicone rubber [24] compared to 0.024 cm^3^(STP)·cm^-3^·atm^-1 ^in water (the latter corresponding to 0.0048 mL/L at normal air). Models as implemented here required the use of a special boundary condition using the stiff-spring method to account for the different solubilities of oxygen in the membrane and in the media; results are presented after recalibrating concentration in the membrane with the partition coefficient *K*_p _= *c*_membr_/*c *(Figure 5). They confirm that, indeed, much better oxygenation can be achieved and that the 'oxygen sandwich' designation [22] is justified as O_2 _can reach the islets from both sides; in fact, under most conditions, a much larger flux is coming from the bottom through the membrane than from the top through the aqueous media. It should be noted that because in such membranes carbon dioxide tends to have an even higher permeability than oxygen [24], in certain cases, it might reach undesirably elevated or undesirably low levels (depending on the outside concentrations).

### Perifusion chamber with flowing media

A model of a perifusion chamber was implemented because perifusion studies are now routinely used to assess islet quality and function as they allow the dynamic measurement of the glucose-stimulated insulin release (GSIR) [54,63-65] through the continuous monitoring of the insulin (and/or other metabolic products) released by islets placed in a perifusion column and exposed to varying levels of incoming glucose solutions. Because of the flowing media, this requires a true multiphysics approach to account for oxygen transport due to both diffusive and advective transfer. Furthermore, additional dynamics was also introduced by allowing the oxygen consumption to increase with the increasing metabolic demand imposed by the presence of higher glucose concentration, when islets are attempting to increase their insulin output. Here, an approximate doubling of the consumption rate was assumed in islets when going from low (3 mM) to high (11 mM) glucose concentrations (eq. 3) – a value in acceptable agreement with the average increase observed in rat islets by Longo and co-workers [53] or, more recently, in human islets by Sweet and co-workers [46] and also showing some correspondence to the increased proinsulin and total protein synthesis in islets in response to increasing glucose levels [66]. As Figure 7 and additional file 6 show, the additional stress of increased metabolic demand might cause increased cell death, if sufficiently prolonged, due to the limited availability of oxygen; hence, straining of avascular islets by exposing them to high glucose levels for long periods of time might expose them to additional risks. Whereas the optimal glucose concentration for islet culture seems to be around 10 mM for rodent islets, it seems to be around 5 mM (90 mg/dL) for human islets [7]. The relative ease of extending the present model not only to arbitrary geometries, but also to complex, multiphysics problems is an important advantage.

## Conclusion

In conclusion, various exploratory cellular-level models for the oxygen consumption of avascular pancreatic islets with physiologically relevant geometries have been implemented and used for simulations; they allow the generation of intuitive, easy to interpret colour-coded figures and animations. Results of the computational models are, as a first estimate, in good quantitative agreement with existing experimental evidence, and they confirm that during culture, hypoxia is often a problem for non-vascularised islets leading to necrosis, especially in the core region of larger islets. The present exploratory calculations can be relatively easily extended to various other geometries or to more complex physical problems. Such *in silico *models should be particularly useful not only to improve the design of cell culture and even cell transplant (i.e., bioartificial pancreas-type) devices, but also to increase the viability and functionality of isolated pancreatic islets, which is of crucial clinical relevance for islet transplantation, and to clarify the mechanism of hypoxia-induced necrosis in avascular tissues in general.

## Competing interests

The author declares that they have no competing interests.

## Authors' contributions

PB is the only author.

## Supplementary Material

Additional file 1 **Animated gif file viewable with an internet browser corresponding to Figure **1 Click here for file

Additional file 2 **Animated gif file viewable with an internet browser corresponding to Figure**2. Click here for file

Additional file 3 **Animated gif file viewable with an internet browser corresponding to Figure**3. Click here for file

Additional file 4 **Animated gif file viewable with an internet browser corresponding to Figure **4. Click here for file

Additional file 5 **Animated gif file viewable with an internet browser corresponding to Figure **5. Click here for file

Additional file 6 **Animated gif file viewable with an internet browser corresponding to Figure **7. Click here for file
