# Supplementary figures and images for: FEM-based oxygen consumption and cell viability models for avascular pancreatic islets
Source: Theor Biol Med Model. 2009 Apr 16;6:5. doi: 10.1186/1742-4682-6-5 (PMC2678100; doi:10.1186/1742-4682-6-5)

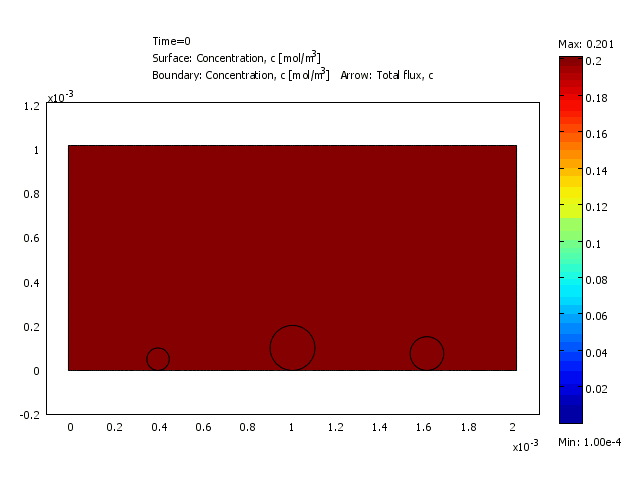

Supplement: Additional file 1 — Animated gif file viewable with an internet browser corresponding to Figure 1 [file 1742-4682-6-5-S1.gif]

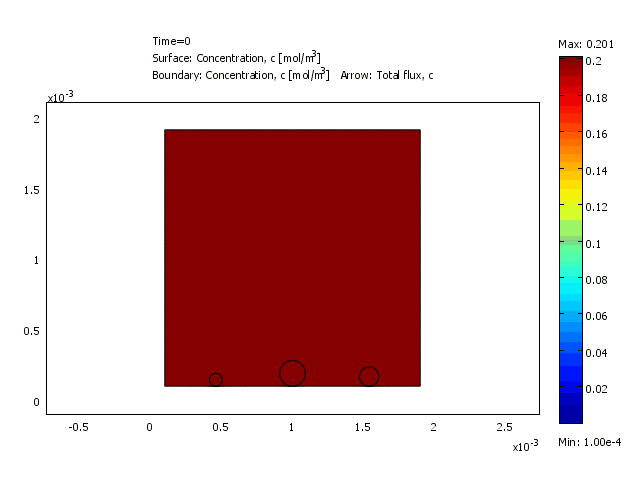

Supplement: Additional file 2 — Animated gif file viewable with an internet browser corresponding to Figure2. [file 1742-4682-6-5-S2.gif]

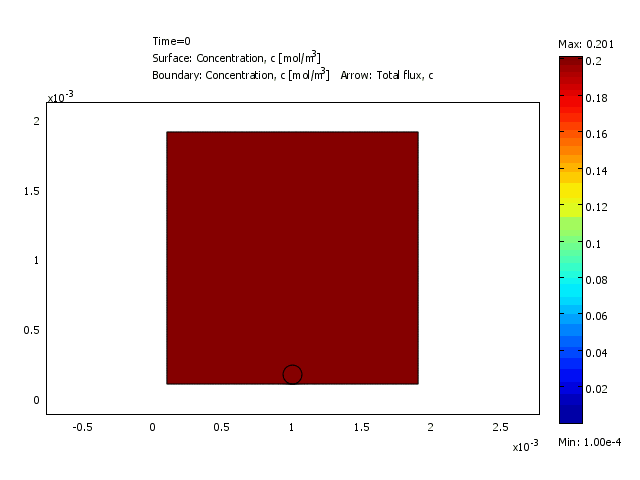

Supplement: Additional file 3 — Animated gif file viewable with an internet browser corresponding to Figure3. [file 1742-4682-6-5-S3.gif]

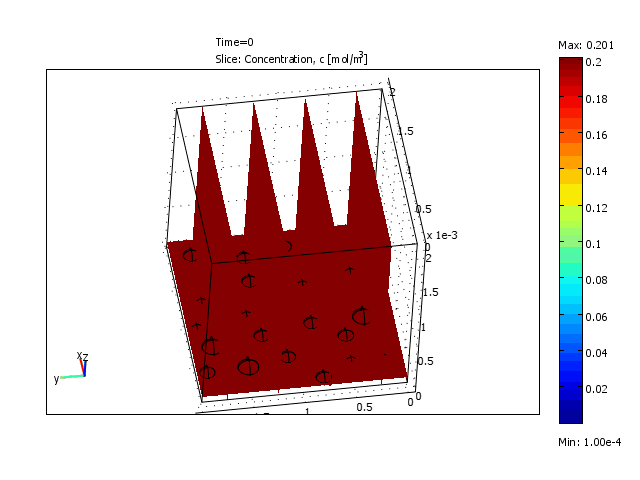

Supplement: Additional file 4 — Animated gif file viewable with an internet browser corresponding to Figure 4. [file 1742-4682-6-5-S4.gif]

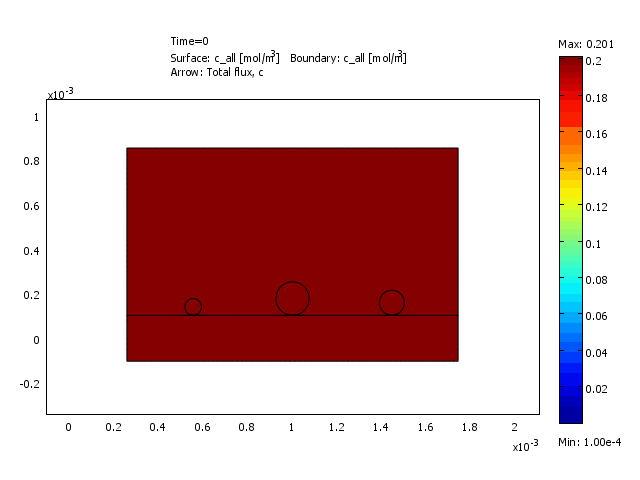

Supplement: Additional file 5 — Animated gif file viewable with an internet browser corresponding to Figure 5. [file 1742-4682-6-5-S5.gif]

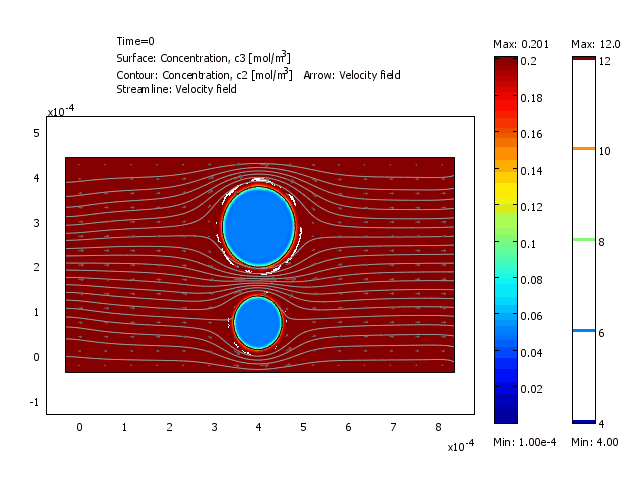

Supplement: Additional file 6 — Animated gif file viewable with an internet browser corresponding to Figure 7. [file 1742-4682-6-5-S6.gif]
